# Intelligence

**Published:** 1826-09

**Authors:** 


### INTELLIGENCE.

### MONTHLY REPORT OF PREVALENT DISEASES.

The last month has presented considerable variations of atmospherical temperature, but by no means so great as that which immediately preceded it. Biliary derangement continues to be by far the most prevalent complaint, and it still assumes the form of diarrhœa, rather than cholera. Upon the whole, the season has, up to the present period, been healthy, and the great majority of the cases we have witnessed have speedily yielded to the exhibition of ten grains of the Hydrargyrum cum[illegible] at night; or, where the purging has been severe, of this combined with a gr[illegible]pium. When these remedies have disagreed, which has very seldom been th[illegible], relief has generally been afforded by ten grains of Rhubarb, with a like quantity of Aromatic Confection, in an ounce of mint-water. In a few cases, the biliary affection has assumed the form of Icterus, requiring and yielding to the usual remedies.

We have seen during the month one case of Delirium Tremens: it was cured by Opium at night, and Ammonia during the day.

Scarlatina has been very prevalent.

It is, perhaps, worthy of remark, that a patient in fever, who was apparently doing well,—having, on the twelfth day, a clean tongue, and pulse at ninety and natural, without any bad symptom whatever,—was very much frightened during the thunder-storm on the evening of the 25th, sunk very rapidly, and died in twelve hours. He was so much afraid of thunder, even when in health, as to be purged by it.

*Scottish Universities.*—The schism which has so long existed between the Senatus Academicus of Edinburgh and their patrons, the Town Council (or magistrates of the city), and of which some notice was taken in former Numbers of this Journal, (October 1824, and January 1825,) has at length terminated in the appointment, by the Crown, of a commission of inquiry, to investigate and report upon the constitution and direction of that and of all the other Scottish universities. This prerogative of the King has not been exercised for upwards of a century, though in ancient times such commissions (or royal visitations, as they are called,) have frequently been issued. The present commission consists of nineteen persons, among whom are the Chancellors of the Universities of St. Andrew's, Glasgow, and Aberdeen, the highest law authorities of Scotland, and the Moderators of the last and present General Assembly of the Scotch Church. The selection of commissioners has certainly been made with great judgment, and cannot fail to obtain the fullest confidence of the country. We are informed that they were to hold their first sitting in the College of Edinburgh, on the last day of August; and much importance is attached, in the Scottish metropolis, to their labours.

---

*Hunterian Museum.*—We have received the following letter with regard to the admission to the Hunterian Museum :—

"Sir,—I perceive that, by a resolution of the Board of Trustees of the Hunterian Museum, admission to that collection is granted to every person presenting a written introduction from a member of the College of Surgeons in London, or a Fellow of the College of Physicians in London; thus excluding from the privilege the whole body of Licentiates, more numerous than the Fellows, and equal to them in medical education,—whose skill and acquirements have been ascertained by examinations similar to their own, and who have been acknowledged equally qualified for medical practice with the Socii themselves.

Why this mark of degradation has been affixed to a class of meritorious individuals, maintaining a respectable station in society, it may be difficult to imagine ; whilst the privilege of introducing visitors is granted to the great body of general practitioners and accoucheurs, to persons keeping retail druggist's shops, and even to the most disreputable quacks, who pollute the columns of our newspapers with their obscene advertisements; for it is well known that individuals of this description are members of the College of Surgeons. But it surely behoves the Licentiates in the metropolis to rescue themselves from this disgrace,—to represent to the trustees the injustice of thus excluding them from the opportunity of visiting the museum intended for the use of the *whole profession,* unless they submit to obtain by favour, (perhaps from an individual of inferior rank to themselves in the profession,) that which should have been conceded of right.

In bringing this subject before the profession, in the pages of your Journal, you may probably be the means of inducing some of the older Licentiates, deservedly possessing much influence, to take up the matter, and procure for their degraded brethren an equality of right, at least, with other practitioners.

*August* 21*st*, 1826. A LICENTIATE."

This letter proceeds upon the assumption that the trustees have unlimited power to admit whom they please. If this be correct, there can be but one opinion of the transaction : if they have not the power, it behoves them to say so. We should like to hear what the Censors have to offer in explanation; for explanation is unquestionably required.

---

*Attendance on Lectures on Midwifery.*—We understand that, after the 1st of January, 1827, the Company of Apothecaries mean to require of candidates for a licence, certificates of having attended two courses of Lectures on Midwifery.

---

*Prussic Acid.*—As the taste for physiological experiments seems to gain ground, and may lead many to try the effect of prussic acid on animals, I am desirous that you should caution students, through the medium of your Journal, how they expose themselves to the influence of the emanations of animals destroyed by that powerful agent, when in the act of dissecting them. On two occasions, within the last two months, but more especially after demonstrating to a few medical friends, this week, the singularly striking effects of prussic acid on a female cat, to which fifteen drops of the medicinal acid had been given, and having been exposed during the dissection, for upwards of two hours, to the vapours of the diffused agent, which had instantaneously destroyed the animal, and appeared to have penetrated every part of its body, I experienced very unpleasant symptoms, such as a slight vertigo and incapability of collecting my thoughts; invincible drowsiness; prostration of strength; yawning; nervousness, with a quick and feeble pulse; anorexia; sinking at the heart; a dread of imaginary evils; and a total want of inclination to act in any way, either bodily or mentally. These disagreeable sensations lasted, more or less, throughout the day, the following night, and the succeeding morning, notwithstanding the use of stimuli and wine; and only gave way, and eventually disappeared, on drinking three cups of strong coffee, with some carbonate of ammonia.

The latter circumstance induces me to remind you, that the medical press have done me injustice in constantly quoting Dr. MURRAY as the person who suggested the administration of ammonia to correct the mischievous effects of prussic acid, and keeping my humble claims out of sight; although I had, long before that gentleman, published the result of my observations in the second edition of my Treatise on Prussic Acid, which went to prove the efficacy of liquid ammonia in obviating the ill effects of an over-dose of the acid, or in restoring animals to which a strong dose of it had been purposely administered.

My observations were published in 1820, and Mr. Murray's paper did not appear until the year after, if not later. (*Note from* Dr. GRANVILLE.)

*Grafton-street, Bond-street; August 20th*, 1826.

---

*Transfusion.*—As a firm believer in the general utility of the operation of Transfusion, when cautiously performed, I feel myself called upon to make a few remarks upon the case which has been so candidly related in the last Number of your Journal; and in doing this, I beg leave to state that I am actuated by no other feeling than a desire to rescue from unmerited obloquy a remedy which promises so much as the operation in question.

The case has gone forth to the world as a *failure*, and so it unquestionably is; but your readers will at once perceive that it is the failure of an operation very different to that which has been recommended, and practised by Dr. BLUNDELL and myself.

The first objection which I have to urge against the operation, as performed in the present instance, is the opening of the jugular vein. It is said (and I have heard this stated by a gentleman who made some experiments on the subject,) that a much smaller quantity of air will destroy life, if injected into the jugular vein, than if it be introduced into a vein of the extremities. This experiment, however, I have never repeated myself, and cannot therefore speak from personal experience.

That air was in the present case thrown into the vein, is, I conceive, a fact which cannot for one moment be doubted: it was proved by the post-mortem examination; and, if we turn our attention to the apparatus used for the injection of the blood, we shall see that it was utterly impossible for it to be otherwise. If I rightly comprehend it, there was an adapting tube introduced into the jugular vein, and through this tube the blood was passed by means of the syringe. Now what is the *unavoidable* consequence of such an arrangement? This small tube must of necessity be full of air, and therefore, at each introduction of the nozzle of the syringe, this quantity must at any rate be forced into the vein; and, although the tube was small, and consequently the proportion of air thrust in at each injection must be small also, yet it is to be recollected that this operation was repeated sixteen times in the short space of twenty minutes; so that, provided there were no *accidental* admission of air, we see there was a *necessary* introduction of sixteen of these tubes full; an occurrence which, I apprehend, is quite sufficient to account for the failure of the operation.

The small size of the syringe is another, and to me a very strong, objection; for it is much more difficult to introduce the blood in a regular and equable stream, than with a larger one. There is another convenience, too, attending the use of a larger syringe, for it enables you to take the further precaution to prevent the admission of air: it is my practice not only to expel the air in the usual manner before I commence one injection, but, in order to avoid the possibility of throwing in any that may by chance have been left behind, I invariably stop before the instrument is perfectly empty, throwing away what remains.

In all cases of desperate hemorrhage, the veins at the bend of the arm are in a collapsed state, and therefore are not readily seen, but I believe it will be found, if one of them be laid bare, and an opening made into it, that it will readily dilate sufficiently to admit the beak of the syringe. This was the case in my last patient: the vein appeared like a small cord, and comparatively empty.

Let it not, however, be supposed that we entertain the chimerical hope of having found a remedy which will invariably be successful: it is a well-known fact, that the most approved and established operations of surgery do occasionally fail. All that we are contending for is this: that it is a remedy from which we may expect much benefit in cases of excessive exhaustion from hemorrhage; and the proportion of successful cases is such, that we can appeal to them with some degree of confidence in the support of our opinion. Mr Jewel, in common with others of the profession, seems to doubt the utility of injecting a few ounces of blood into the system of a patient who has lost several pounds. Now I am most decidedly of opinion, (and my opinion has not been hastily formed,—it is founded upon actual experiment and observation,) that the success of the operation will be greatly hazarded if too much blood be thrown in. The vascular system can no more bear to be suddenly filled than to be suddenly emptied; and therefore, when a sufficient quantity has been injected to relieve the state of syncope under which the patient has been suffering, I consider it much better to stop the operation, and not run the risk of oppressing the system by injecting too much. After we have succeeded in conveying a certain degree of power to the circulation, we may safely trust to the recruiting powers of the digestive organs; the intention of the operation being by no means the introduction of the same quantity of blood into the blood-vessels which they contained previous to the hemorrhage. Of course, no definite rules can be laid down as to quantity; we must be guided entirely by its effects.

It may not be uninteresting to some of your readers to be informed, that Mr. Bingham, of Manchester, has lately performed the operation with complete success. An account of the case is given in the Edinburgh Journal of Medical Science. (*Note from* Mr. Waller.)

111, *Aldersgate-street*; *August* 1*st*, 1826.

---

*New Division of the Thermometer*.—The difference observed at Melville Island between the degrees of temperature indicated by the thermometers, when placed in the same circumstances on the ice, that were taken out by the north-west expedition ships in 1820, has led Lieutenant A. M. Skene, R.N. to endeavour to remedy this error of thermometers; for which he has invented a new division of the thermometrical scale, taking the fusion of two solid bodies, instead of taking the evaporation and the freezing of a fluid, as has hitherto been done; for we cannot unite at pleasure circumstances conducive to the evaporation of a fluid at a fixed degree of temperature: whereas, on the contrary, the fusion of bodies is only determined by the affinity of the molecules of the bodies the one for the other, and for caloric, and depends on no other cause. Therefore, Mr. Skene takes the difference of temperature between the fusion of ice and the fusion of frozen mercury, as a thermometrical *unity*, taking care that these two bodies are perfectly pure, which unity he denominates a *degree*, and subdivides this degree into one hundred *minutes* or parts, in imitation of the new division of the terrestrial circle. The fusion of ice will preserve the station it does with nearly all nations that make use of thermometers, and will be designated by the sign 0; it will also divide heat from cold, which have as much two separate existences as any two sensations, and which are attended with the same natural phenomena. It is a curious circumstance, but one of no importance, that 360 degrees, which is the division of a circle, is also the greatest probable heat, using Mr. Skene's scale as according to Mr. Wedgewood's Pyrometer. From observing that the atmospheric heat ranges as much above zero when you approach the Equator, as it ranges below zero when you approach the Poles, Mr. S. is induced to believe that there is the same extent or circle of cold of 360 degrees, as there is of heat. The positive sense, or ascending the scale, will be marked by the sign +; whilst the negative sense, or descending the scale, will be designated by the sign —. This scale will have the advantage of indicating the temperatures of the fusion of bodies; the least fusible by small numbers. It also is a decimal scale. Between the fusion of ice and the boiling of water, there will be but 2.50, which may be indicated by 2.5+0. Zinc melts at 9.+0; spermaceti melts at 1.+0; frozen mercury fuses at 1.—0, &c. &c. These numbers will be more easy to retain than those which are actually in use.

It is true that the graduating thermometers will become more difficult, and cannot be confided but to artists instructed, or those who possess a standard thermometer; but, far from there resulting any inconvenience, it will tend to do away with the multitude of badly-divided and incorrect instruments, which never agree together in the same circumstances, and to which faith cannot be given when observations of importance are required. These instruments, graduated according to the method of Mr. S., will necessarily be of accord in whatever place they may have been made. (*Note from* Mr. Skene.)

---

*New Work on Diseases of the Skin.*—We are happy to learn that Dr. A. T. Thomson is preparing for publication a new edition of Dr. Bateman's useful Synopsis. No one can have turned his attention to cutaneous affections, without being forcibly struck with the extreme difficulty of conveying a distinct and satisfactory idea of the various forms of eruption by any verbal description, however graphic. Willan was fully aware of this when he published his plates; but unfortunately the expence of this work, as originally published by Willan, and subsequently by Bateman, is such as to place it beyond the reach of most students. These disadvantages, we understand, are to be obviated in the edition now preparing, as each disease will be depicted as well as described, while the expence will be extremely moderate.

---

*Anatomy.*—Mr. Tuson, who was lately House-surgeon to the Middlesex Hospital, has in the press a System of Anatomy, illustrated by plates on a peculiar construction. Mr. Tuson's method is extremely ingenious, consisting of lithographic representations of the bones, over which are laid the muscles, of their relative sizes and shapes. The limb is as it were built up in this way, and the parts may be again raised in succession, from the integuments to the bone. The fasciculus already published contains the muscles of the anterior and posterior parts of the thigh, leg, and foot; and it is proposed to follow it by other fasciculi, embracing the muscles of the other parts of the body. It is also in contemplation to give a Supplement on a similar plan, containing the anatomy of the arteries, veins, nerves, and lymphatics, the abdominal and thoracic viscera, the brain, eye, ear, the fœtal circulation, the secretions of bile, urine, semen, &c. Should these objects be accomplished in a manner corresponding to the specimen already published, it will form a work of very great utility to the student, and be highly creditable to the author.

### METEOROLOGICAL JOURNAL,

***From July 20th, to August 20th, 1826.***

By Messrs. Harris and Co. Mathematical Instrument Makers, 50, High Holborn.

| July | Moon. | Rain gauge. | Thermom. | | | Barometer. | | De Luc's Hygrom. | | Winds. | | Atmospheric Variations. | | |
|---|---|---|---|---|---|---|---|---|---|---|---|---|---|---|
| | | | 9 A.M. | MAX. | MIN. | 9 A.M. | 10 P.M. | 9 A.M. | 10 P.M. | 9 A.M. | 10 P.M. | 9 *a.m.* | 2 *p.m.* | 10 *p.m.* |
| 20 | | .10 | 65 | 73 | 60 | 29.90 | 29.65 | 63 | 72 | W | SW | Fine | Rain | Rain |
| 21 | | | 67 | 69 | 49 | 29.47 | 29.52 | 64 | 75 | WSW | SW | Cloudy | Fair | Fair |
| 22 | | | 65 | 69 | 65 | 29.61 | 29.80 | 70 | 84 | NE | N | Fair | — | Rain |
| 23 | | | 56 | 61 | 53 | 29.82 | 29.89 | 85 | 86 | N | NNEv | Rain | Rain | — |
| 24 | | .82 | 58 | 69 | 56 | 29.88 | 29.96 | 92 | 82 | NE | NE | Clo. Ra. | Fair | Fair |
| 25 | | | 65 | 72 | 56 | 29.99 | 30.04 | 66 | 70 | N | E | Fair | Fine | — |
| 26 | ☾ | | 65 | 72 | 52 | 30.10 | 30.15 | 72 | 68 | ENE | E | Fine | — | — |
| 27 | | | 64 | 71 | 56 | 30.16 | 30.12 | 61 | 68 | ENE | ESE | — | — | Starli. |
| 28 | | | 67 | 73 | 57 | 30.09 | 30.00 | 68 | 72 | ENE | ESE | — | — | — |
| 29 | | | 70 | 77 | 60 | 29.94 | 29.90 | 63 | 64 | WSW | SW | — | — | — |
| 30 | | | 72 | 80 | 65 | 29.92 | 29.88 | 60 | 55 | SW | SSE | — | — | — |
| 31 | | | 80 | 84 | 69 | 29.83 | 29.80 | 54 | 54 | SSW | NE | Cloudy | — | — |
| Aug. | | | | | | | | | | | | | | |
| 1 | | | 74 | 80 | 63 | 29.87 | 29.91 | 62 | 69 | NE | ENE | Fair | — | — |
| 2 | | | 68 | 78 | 63 | 29.84 | 29.78 | 80 | 76 | E | E | Da.&Cl. | Fair | Fair |
| 3 | ● | | 67 | 73 | 55 | 29.76 | 29.80 | 83 | 92 | NE | E | Rain | Rain | R.T.&L |
| 4 | | .135 | 62 | 73 | 58 | 29.81 | 29.86 | 84 | 78 | NE | NE | Cloudy | Fair | Starli. |
| 5 | | | 62 | 69 | 59 | 29.85 | 29.85 | 84 | 79 | NE | S | Rain | — | — |
| 6 | | .27 | 68 | 68 | 63 | 29.90 | 29.98 | 70 | 63 | NW | W | Cloudy | — | — |
| 7 | | | 71 | 76 | 62 | 30.02 | 29.99 | 62 | 65 | W | W | Fair | Fine | — |
| 8 | | | 68 | 78 | 62 | 29.94 | 29.85 | 71 | 65 | WSW | W | Fine | — | — |
| 9 | | | 68 | 76 | 61 | 29.83 | 29.82 | 66 | 71 | NE | E | — | — | Cloudy |
| 10 | ☽ | .9 | 67 | 72 | 60 | 29.81 | 29.76 | 72 | 71 | E | SSE | Cloudy | — | — |
| 11 | | .30 | 63 | 65 | 55 | 29.71 | 29.77 | 75 | 79 | SW | NWv. | Rain | Rain. | Fair |
| 12 | | | 65 | 70 | 55 | 29.85 | 30.01 | 67 | 59 | W | W va. | Fair | Fine | Starli. |
| 13 | | | 67 | 72 | 56 | 30.05 | 29.91 | 64 | 61 | S | SE | — | — | — |
| 14 | | | 71 | 76 | 57 | 29.79 | 29.90 | 62 | 68 | SW | WSW | — | — | — |
| 15 | | | 64 | 73 | 61 | 29.92 | 29.83 | 70 | 70 | SW | SW | — | — | — |
| 16 | | | 64 | 70 | 58 | 29.81 | 29.85 | 79 | 72 | W | W | Rain | Fair | Cloudy |
| 17 | | | 63 | 73 | 65 | 29.91 | 80.08 | 72 | 70 | WSW | WSW | Cloudy | — | Fine |
| 18 | ○ | | 73 | 78 | 62 | 30.14 | 30.18 | 69 | 68 | WSW | SW | Fair | — | — |
| 19 | | | 67 | 79 | 62 | 30.16 | 30.03 | 69 | 70 | SW | E | — | — | — |

The quantity of Rain fallen in the month of July, was 1 inch and 16.100ths.

